# “Blebbing” of a Gold Nanocluster by Dioxygen Insertion into Thiolate Staples during Self-Photooxidation

**DOI:** 10.1021/acs.jpca.5c06629

**Published:** 2025-12-12

**Authors:** Serah Essang, Alexander Greer

**Affiliations:** † Department of Chemistry, 2037 Brooklyn College, Brooklyn, New York 11210-2814, United States; ‡ Ph.D. Program in Chemistry, The Graduate Center of the City University of New York, New York, New York 10016, United States

## Abstract

In our work with a Au thiolate nanocluster (Au_20_(SG)_16_, where SG is the tripeptide glutathione), we noticed that it underwent a self-photooxidation reaction in the presence of white light and oxygen. We now report on mechanistic studies using photophysical, photochemical, theoretical, and indirect trapping methods. We find rapid total quenching of singlet oxygen (^1^O_2_) by ground-state Au_20_(SG)_16_, with evidence of dioxygen insertion into the nanocluster. Supported by analyses with IR, ESI-MS, and density functional theory, we propose the formation of Au–O–O–SG bonds in the Au nanocluster. The expansion of the staple motif from dioxygen insertion is attributed to heightened lability and blebbing (a protrusion) arising from the O–O group. We then demonstrated that the self-photooxidized Au_20_(SG)_16_ undergoes oxygen-atom transfer to a phosphine trap in the dark.

## Graphical abstract

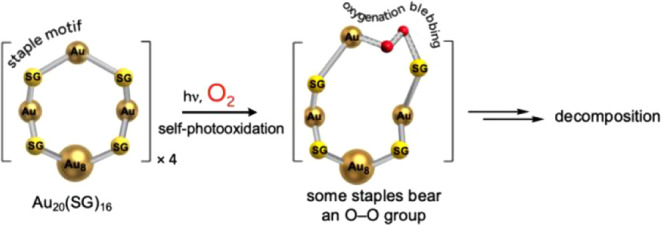

## Introduction

Au thiolate nanoclusters continue to strike the interest of physical chemists, where they can vary in the size and number of Au atoms and types of thiolate ligands.^1–4^ Scheme 1A shows the Au nanocluster system that we were interested in, namely, Au_20_(SG)_16_, which contains a Au_8_ core and four staple motifs of −(SG)–Au–(SG)–Au–(SG)–Au–(SG)–, where (SG) is an abbreviation for the tripeptide glutathione. It can be noted that Au thiolate nanoclusters were previously studied using both aliphatic and aromatic thiolates.^5–7^ Furthermore, Au thiolate nanoclusters can serve as photosensitizers^8–10^ and give rise to reactive oxygen intermediates,^11,12^ including singlet oxygen (^1^O_2_).^13–17^

**Scheme 1:**
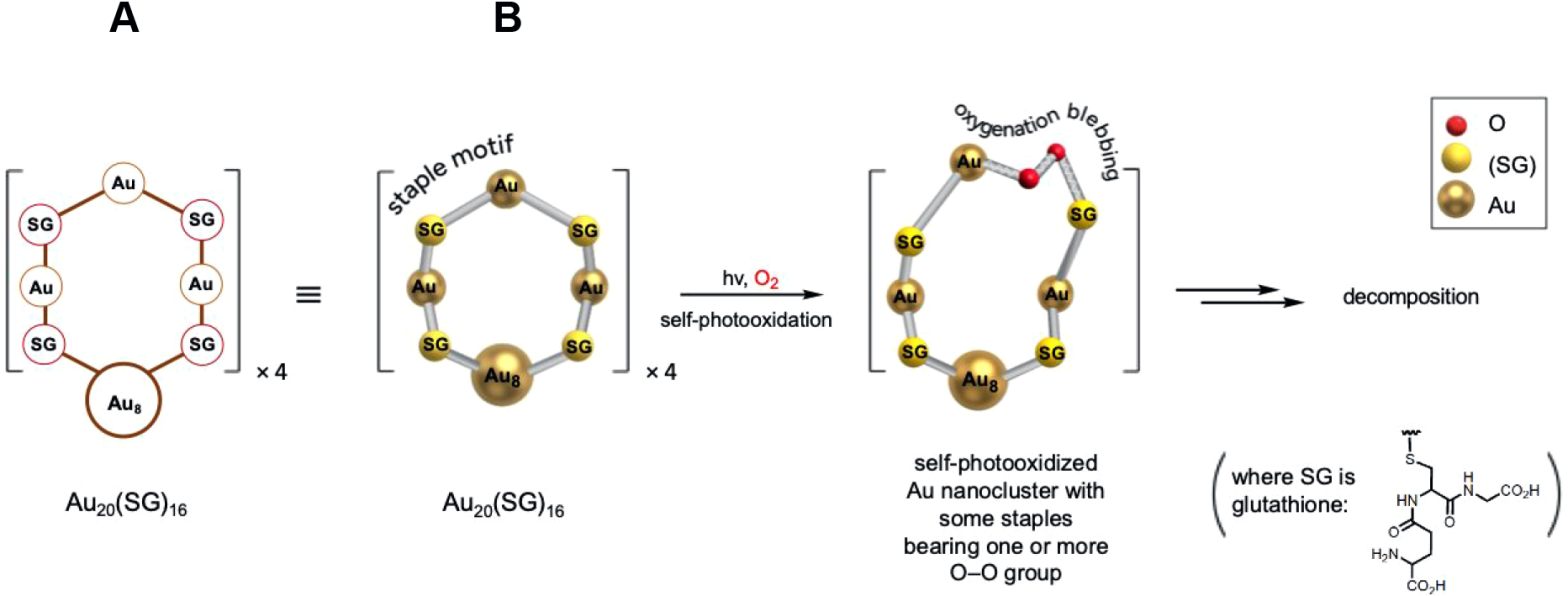
Schematic Showing Au_20_(SG)_16_ as an Abbreviated Structure with a Au_8_ Core and Periphery of Four Staple Motifs, where (SG) is an Abbreviation for the Tripeptide Glutathione^a^

a The thiolate moieties in the glutathione bridging ligands bind to two Au atoms per sulfur atom. The self-photooxidized Au nanocluster can contain staple motifs with one or more O–O group.

As such, Au nanoclusters are useful for photogenerating reactive oxygen intermediates both *in vitro* and *in vivo*.^18–30^ Reports have also examined ligand effects to stabilize or make the nanocluster biocompatible,^31–37^ including the use of intercluster disulfide linkages,^38^ cerium oxide supports, and oxidation reactions.^39,40^ As it stands, there remains a need for mechanistic studies of photooxidation reactions of Au-thiolate nanoclusters.

Indeed, a more complete mechanistic understanding of the consequences of exposure of the Au nanocluster to “self” photogenerated reactive oxygen intermediates is needed. Due to the Au nanocluster’s photosensitizer activity, oxygenation blebbing of Au_20_(SG)_16_ can occur as illustrated in Scheme 1B. “Blebbing” is a term from biology but also fits here due to a protrusion arising in the nanocluster by insertion of an O–O group.

Like other photosensitizers, Au nanoclusters are susceptible to self-photooxidation.^41–43^ By analogy, the self-photooxidation of the sensitizer heptamethine cyanine with ^1^O_2_ led to an allene hydroperoxide^44,45^ and toluidine blue O (TBO) to a peroxyl radical, both with subsequent decomposition (Scheme 2A,B).^46,47^ Also, the self-photooxidation of protoporphyrin IX (PpIX) led to endoperoxides and other peroxides and then decomposition (Scheme 2C).^48^ Self-photooxidation decomposition reactions seem to proceed by peroxy intermediates, such as ROO**•** radicals, ROOH and ROOR species.^48–50^ Thus, we wondered whether the Au_20_(SG)_16_ nanocluster will also self-photooxidize by way of a peroxy intermediate or differ mechanistically from other photosensitizers.

**Scheme 2:**
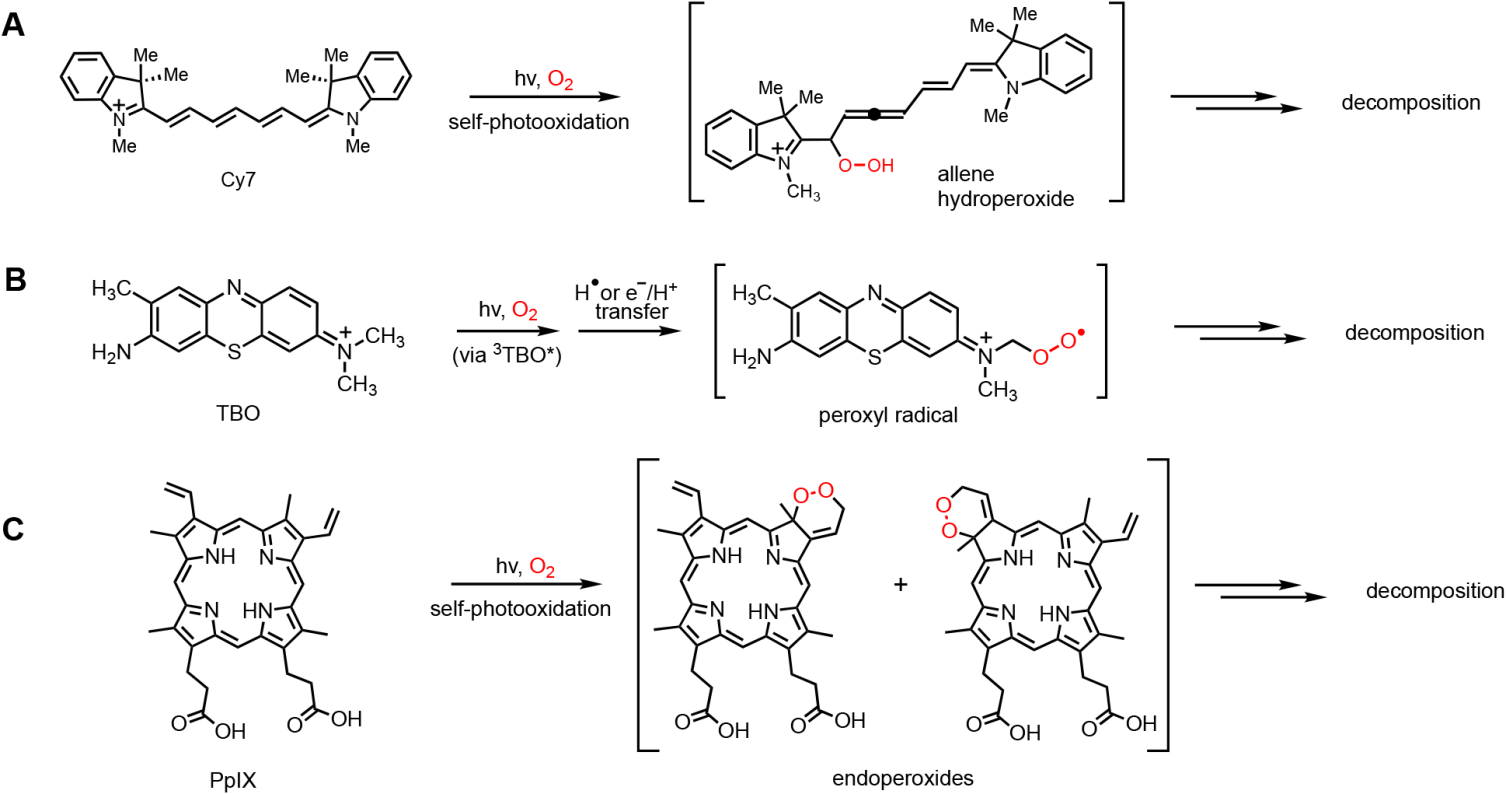
Photosensitizers Undergoing Self-Photooxidation to Reach Peroxide Intermediates Such as Hydroperoxides (ROOH), Peroxyl Radicals (ROO**•**), and Endoperoxides (Six-Membered Ring ROOR) Prior to Decomposition[Fn sch2-fn2]

a Such decompositions are often referred to as photobleaching, although usually ill-defined.

Here, we report on the self-photooxidation of Au_20_(SG)_16_. The results include: (1) detection of the ^1^O_2_ near-IR signal from Au_20_(SG)_16_ by time-resolved techniques and determination of its total quenching rate constant (*k*_T_) with ^1^O_2_, (2) mass spectrometry evidence for the uptake of dioxygen into the nanocluster, (3) IR and DFT evidence point to the existence of a Au–O–O–SG species and not Au persulfoxide, sulfinate ester, or other species, and (4) evidence that the gold peroxide intermediate transfers an O atom to a water-soluble phosphine in a ^31^P NMR peroxide test.

As we will see, results point to oxygenation blebbing of the staples in Au_20_(SG)_16_ in a new staple expansion process by dioxygen insertion. Protrusion on the staple can cause instability and rupture in the nanocluster. A new mechanism for oxidative blebbing of the Au nanocluster is presented, but questions remain on the decomposition products to smaller Au nanoclusters

## Experimental Section

### General Aspects

Acetic acid, acrylamide, aluminum­(III) phthalocyanine tetrasulfonate tetraanion (AlPcS_4_), CH_3_OH, CH_3_CN, CD_2_Cl_2_, CDCl_3_, D_2_O, glycerol, 3-(diphenylphosphino)­benzenesulfonate anion, diphenylsulfide, glutathione (GSH), glycine, hydrogen tetrachloroaurate­(III) hydrate (HAuCl_4_•aq.), NaNO_2_, Na_2_SO_4_, *N*,*N’*-methylenebis­(acrylamide), sodium borohydride (NaBH_4_), trifluoroacetic acid, and *tris*(hydroxymethyl)­aminomethane were used as received from commercial suppliers. Deionized water was obtained from a deionization system. Two photolysis systems were used: (i) 532 nm light from an Nd:YAG Q-switched laser (10 mJ pulse, 5 Hz), and (ii) (400 < λ < 700 nm) light from a metal-halide lamp and a 12 M NaNO_2_ filter solution. HPLC spectra were collected with an eluting solvent system (all solvents contained 0.1% trifluoroacetic acid; a flow rate of 1 mL/min) with a linear gradient of 1% CH_3_CN/H_2_O (*v/v*) that increased to 99% CH_3_CN/H_2_O (*v/v*) at 6 min and then remained constant up to 8 min. UV–visible spectra were collected for Au_20_(SG)_16_ (1 μM) in deionized H_2_O (3 mL) in a quartz cuvette in which spectra were acquired at a scan rate of 200 nm/min. ESI-MS data were collected with mass-to-charge resolution to four decimal places, where experimental masses were in agreement with predicted masses to mass errors within an acceptable range. For ESI-MS, mass error was calculated by subtracting the experimental mass from theoretical or predicted mass and then dividing by the theoretical mass and multiplying by 10^6^ to get the ppm value. ^31^P NMR spectra were proton decoupled and obtained in D_2_O with a 400 MHz Bruker NMR spectrometer, with 256 scans and a spectral width of 49019.60 Hz.

### Synthesis of Au_20_(SG)_16_

Yield (50 mg, 18.5%), purity (97.4%). Based on a literature report,^14^ a solution of HAuCl_4_•aq. (0.25 mmol) in 50 mL methanol was stirred for a few minutes, after which 1.0 mmol of GSH was added to it. This mixture was allowed to cool to 0 °C in an ice bath with stirring for 30 min. An aqueous solution of NaBH_4_ (2.5 mmol in 10 mL) was added to the mixture and stirred for another 1 h. A dark brown precipitate was formed that was then collected and washed with methanol through centrifugation to remove the remaining starting materials. The resulting precipitate was then vacuum-dried to obtain the Au thiolate nanoclusters as a dark-brown powder. This Au thiolate nanocluster sample was then added to 50 mL of methanol and then stirred at 700 rpm at 50 °C for 12 h to remove smaller nanoclusters. The Au thiolate nanocluster solution was allowed to cool and washed several times with methanol by centrifugal ultrafiltration (6000 *g*). The resulting mixture was further purified by polyacrylamide gel electrophoresis (PAGE). Acrylamide monomers with a 94:6 ratio of acrylamide to *N*,*N’*-methylenebis­(acrylamide) were used, with the total amount for the separating and stacking gel being 30 and 3 wt %, respectively. Glycine (192 mM) and *tris*(hydroxymethyl)­aminomethane (25 mM) were used to prepare the eluting buffer. A 20 mg/mL concentration of the Au thiolate nanoclusters was prepared in a 5% (*v/v*) glycerol/water solution. For separation, 0.5 mL of sample solution was loaded onto the stacking gel and eluted for 2 h at a constant voltage of 100 V. This process was carried out in a cold room. The fractions were carefully cut out of the separating gel, crushed, soaked in 2 mL of deionized water, and placed in the refrigerator for 4 h. The suspension of gel lumps was removed with a 0.2 μm pore filter. Then, 1 mL of 2% acetic acid was added to the sample followed by precipitation in 1.5 mL methanol and centrifugation. The resulting precipitate was redissolved in 0.5 mL deionized water and further purified to remove smaller impurities by washing via centrifugal ultrafiltration twice. A filter with a cutoff molecular weight of 3 kDa was used. Figure S1 (Supporting Information) shows an HPLC spectrum of purified Au_20_(SG)_16_; Figure S2 and Table S1 show ESI-MS data, including *m*/*z* 1768.3102 with charge *z* = 5 corresponds to a deconvoluted mass of 8841.5510 in agreement with the predicted mass of cluster ion [Au_20_(SG)_16_ + 5H]^5+^. Similarly, *m*/*z* values with charges *z* = 4 and 3 were found, corresponding to the predicted masses of cluster ions [Au_20_(SG)_16_ + 4H]^4+^ and [Au_20_(SG)_16_ + 3H]^3+^.

### Total Quenching Rate Constant (*k*_T_) Measurements

A laser system was used as previously described,^51,52^ where green light (532 nm) arose in 10 mJ pulses from a Nd:YAG Q-switched laser at 5 Hz. The ^1^O_2_ phosphorescence was detected by using an A H10330A-45 (Hamamatsu Corp.) photomultiplier tube operating at −650 V. This phosphorescence was monitored through a band-pass filter centered at 1270 nm (OD4 blocking, fwhm = 15 nm), in which signals were collected on a 600 MHz oscilloscope. A monoexponential function was then used to fit the output ^1^O_2_ decay curve.

$$I=I_{0}+Ae^{-t/\tau \Delta }$$

In the above equation, *I* represents the final intensity, *I*_0_ is the initial intensity, *t* is time, *A* is the amplitude, and τ_Δ_ is the lifetime of ^1^O_2_. Total quenching rate constants (*k*_T_) were determined by fitting the data to plots of *k*_obs_ vs [Au_20_(SG)_16_] with the following equation:

$$k_{obs}=k_{d}+\left(k_{T}\right)\left[Au_{20}{\left(SG\right)}_{16}\right]$$

in which *k*_obs_ is the observed ^1^O_2_ quenching rate constant and *k*_d_ is the rate constant for deactivation of ^1^O_2_ by the solvent. Lifetime measurements of ^1^O_2_ (τ_Δ_) were measured in D_2_O alone (τ_Δ_ = 65.3 μs) and at different concentrations of Au_20_(SG)_16_. Singlet oxygen lifetimes were also measured at different concentrations of GSH by using AlPcS_4_ (50 μM) as the sensitizer. We selected concentrations of Au_20_(SG)_16_ and GSH to decrease the ^1^O_2_ lifetime (τ_Δ_) by 10–30 μs. *k*_T_ measurements were determined for Au_20_(SG)_16_/^1^O_2_ and GSH/^1^O_2_ reactions in D_2_O at room temperature.

### Self-Photooxidation Procedure

Oxygen-saturated D_2_O solutions containing Au_20_(SG)_16_ (0.1 mM) were irradiated with (400 < λ < 700 nm) light. Photooxidations of GSH (0.1 mM) were also carried out in the presence of AlPcS_4_ (50 μM). The photooxidations were typically carried out to 80% conversion of Au_20_(SG)_16_ and GSH. One milliliter solutions of Au_20_(SG)_16_ (0.1 mM) and GSH (0.1 mM) with AlPcS_4_ (50 μM) were irradiated with (400 < λ < 700 nm) light in oxygen-saturated D_2_O. Phosphine trapping experiments were also carried out: after the self-photooxidation of Au_20_(SG)_16_ or the photooxidation of GSH with AlPcS_4_, 3-(diphenylphosphino)­benzenesulfonate anion **1** (0.04 mM) was added to the solution with mixing, where relative amounts of the corresponding phosphine oxide, 3-(diphenylphosphoryl)­benzenesulfonate anion **2** were compared by ^31^P NMR. Phosphine oxide **2** formation did not occur in the dark, although a trace amount of **2** (1.3%) was present as an impurity prior to the photoreaction. Similar postphotooxidation trapping of fatty acid hydroperoxides and other peroxides with phosphine **1** has been reported previously.^53^ Experiments were also carried out using a chemical source of ^1^O_2_. Au_20_(SG)_16_ (0.1 mM) was mixed with 3-(4-methyl-1,4-epidioxynaphthalen-1­(4H)-yl)­propanoate dianion in D_2_O for 1 h. The reaction temperature was maintained below 15 °C, and the ^1^H NMR spectrum of the mixture was collected every 10 min. The 3-(4-methyl-1,4-epidioxynaphthalen-1­(4*H*)-yl)­propanoate dianion was synthesized in 92% yield with 96% purity (0.37 g). It was precipitated at pH 2 by acidifying with 2 M phosphoric acid, and the precipitate was collected by vacuum filtration over ice to prevent decomposition.

### Computations

All computational calculations were carried out in the gas phase, while following the recommended procedure on the Gaussian-16 software package.^54,55^ M06-2X was used,^56^ and a split-basis set was utilized, where nonmetals were treated with a high-quality Pople-type or Dunning-type basis set, while the transition metal, Au was modeled with the LANL2DZ effective core potential (ECP) basis set. This method was specified by using the GENECP keyword, which enables optimum computational efficiency and accuracy in electronic structure calculations.^57,58^ To find equilibrium geometries during optimizations, the opt keyword was used. Charge transfer effects and electronic degeneracies can sometimes be a challenge, so we used the key word guess = (mix, always) to improve the molecular orbital guess. The SCF = Tight keyword was used to improve numerical stability and convergence. In addition to this, the scf = novaracc keyword was used to enforce a more stringent convergence criterion in our calculations. Finally, to account for diffuse electron densities or strong electron correlation effects in our system, the keyword int­(grid = ultrafine), which refers to an ultrafine integration grid, was utilized to improve the accuracy of numerical integration for exchange-correlation terms.

## Results and Discussion

### Photolysis Studies

First, we studied Au_20_(SG)_16_ and found it to sensitize the formation of ^1^O_2_ and also quench ^1^O_2_ at higher concentrations. For Au_20_(SG)_16_, the total quenching rate constant (*k*_T_) of 1.4 × 10^8^ M^–1^ s^–1^ was measured by plotting ^1^O_2_ lifetime vs concentration (Figure 1A). We sought to examine the quality of our *k*_T_ value due to Au_20_(SG)_16_’s dual role as a sensitizer and quencher of ^1^O_2_. Thus, we analyzed the Au_20_(SG)_16_/^1^O_2_ reaction in the presence of AlPcS_4_ (50 μM) as a cosensitizer and found only a modest 18% greater of 1.7 × 10^8^ M^–1^ s^–1^. Consequently, we can confidently bracket the *k*_T_ for Au_20_(SG)_16_ at ∼1–2 × 10^8^ M^–1^ s^–1^. Furthermore, the *k*_T_ for the GSH was 9.1 × 10^6^ M^–1^ s^–1^ (Figure 1B), which indicates that ^1^O_2_ quenching by Au_20_(SG)_16_ was 11- to 22-fold higher than GSH itself, indicating that ^1^O_2_ quenching is about proportional to the glutathione content in the Au nanocluster.

**Figure 1:**
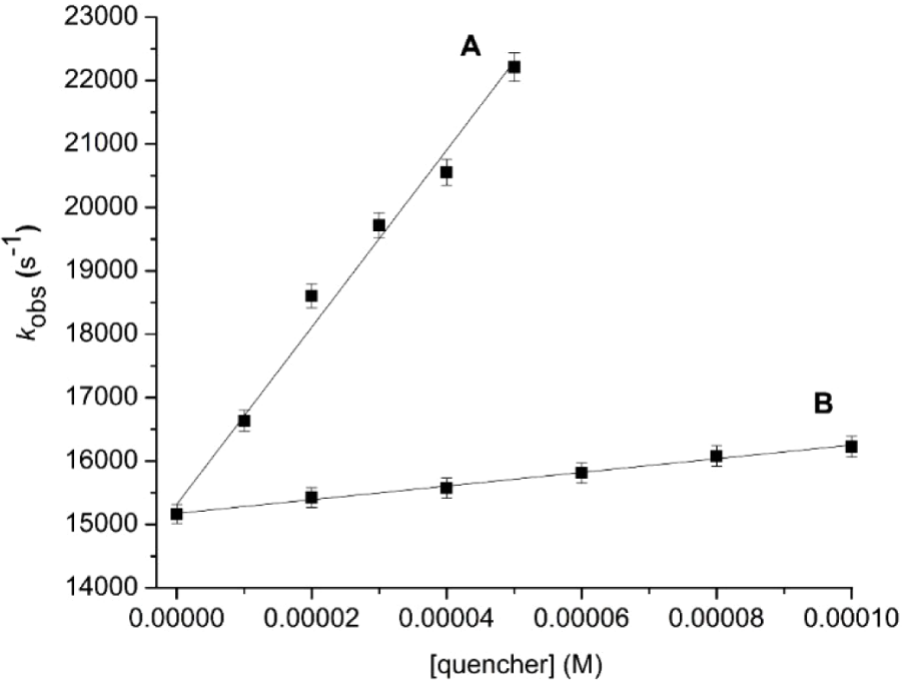
Total quenching rate constant (*k*_T_) plots of ^1^O_2_ in D_2_O at 25 °C. The *k*_obs_ (s^–1^) is shown in relation to molarity of (A) Au_20_(SG)_16_ quencher alone and (B) GSH quencher in the presence of AlPcS_4_ (50 μM). Data were collected four times and then used to determine the standard deviation.

Next, we examined the self-photooxidation of Au_20_(SG)_16_ with reactions carried out in oxygen-saturated D_2_O with (400 < λ < 700 nm) light exposure using ESI-MS and UV–vis spectroscopy. First, ESI-MS detected five cluster ions indicating the uptake of oxygen into Au_20_(SG)_16_: [Au_20_(SG)_16_O_14_ + 14H]^14+^, [Au_20_(SG)_12_O_2_ + 14H]^14+^, [Au_2_SG_8_O_3_ + 4H]^4+^, and [Au_3_(SG)_6_O_10_ + 3H]^3+^ and [Au_3_(SG)_6_O_11_ + 3H]^3+^ (Table 1 and Figure S4). The peaks found at *m*/*z* 649.4801 and 547.0269 are presumed to be *z* = +14 and attributed to cluster ions [Au_20_(SG)_16_O_14_ + 14H]^14+^ and [Au_20_(SG)_12_O_2_ + 14H]^14+^; their error bounds were adequate, but their abundances were low due to poor ionization efficiency. The chemical formulas of the oxygenated nanoclusters in Table 1 are tentative. Longer retention times are seen with higher oxygen-to-Au ratios, e.g., [Au_20_(SG)_16_O_14_ + 14H]^14+^ and [Au_20_(SG)_12_O_2_ + 14H]^14+^ (3.7–3.9 min), [Au_2_(SG)_8_O_3_ + 4H]^4+^ (2.9–3.4 min), and [Au_3_(SG)_6_O_11_ + 3H]^3+^ and [Au_3_(SG)_6_O_10_ + 3H]^3+^ (5.7–7.3 min). These oxygenated nanoclusters have much longer retention times compared with the native cluster ion [Au_20_(SG)_16_ + 4H]^4+^ (0.9–1.0 min). While uptake of oxygen into the photooxidized nanoclusters was evident, their structures were not. Second, UV–visible spectra were collected for Au_20_(SG)_16_ prior to and after self-photooxidation (Figure 2). Two main absorptions are observed: an absorption at 380–450 nm (denoted as Au_s‑sp1_) and an absorption at 570 nm (denoted as Au_s‑sp2_) corresponding to what is known as the Au_6sp_ intraband transitions. These two UV–visible absorptions decreased upon self-photooxidation, which we attribute to the degradation of Au_20_(SG)_16_ into smaller nanoclusters. However, the UV–visible data did not provide much insight into chemical bonding of the self-photooxidized nanocluster, leading us to IR spectroscopic studies.

**Table 1:** ESI-MS Data of Au_20_(SG)_16_ Prior to and after Self-Photooxidation in D_2_O^a^

	Cluster ion	Experimental *m*/*z*	Experimental Monoisotopic Mass	Calculated Monoisotopic Mass	Mass Error (*ppm*)
unoxidized	[Au_20_(SG)_16_ + 4H]^4+^	2210.1401	8836.5312	8836.5471	3.69
after photooxidation	[Au_20_(SG)_16_O_14_ + 14H]^14+^	649.4801	9078.6192	9078.6167	0.28
	[Au_20_(SG)_12_O_2_ + 14H]^14+^	547.0269	7644.2744	7644.2330	5.40
	[Au_2_(SG)_8_O_3_ + 4H]^4+^	720.6218	2878.4580	2878.4318	9.10
	[Au_3_(SG)_6_O_11_ + 3H]^3+^	870.7892	2609.3457	2609.3466	0.35
	[Au_3_(SG)_6_O_10_ + 3H]^3+^	865.1126	2592.3159	2592.3439	10.8

a Concentration of Au_20_(SG)_16_ was 0.1 mM.

**Figure 2:**
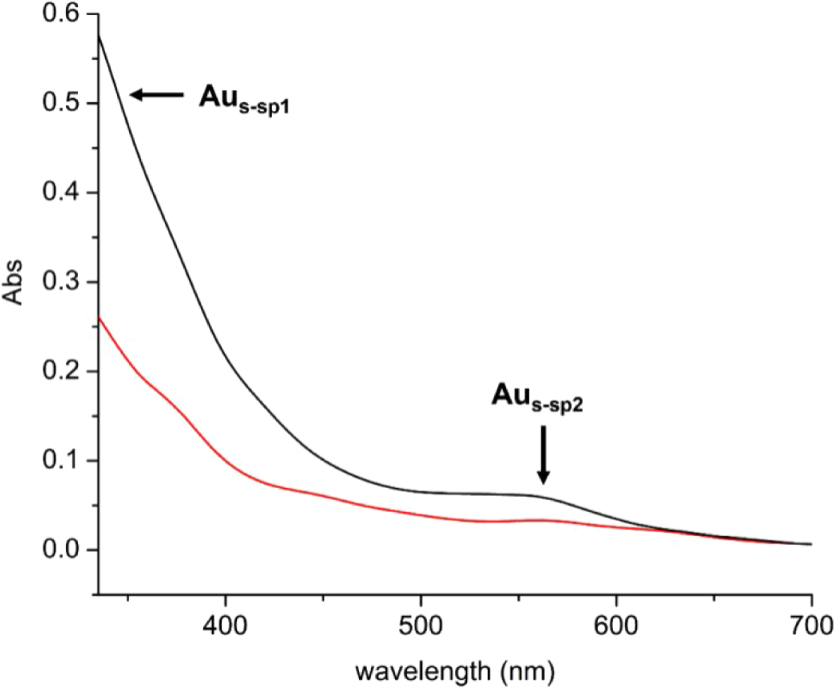
Absorption spectra in deionized H_2_O of Au_20_(SG)_16_ prior to photooxidation (black trace) and Au_20_(SG)_16_ after photooxidation (red trace). We observed a decrease in the absorbance of both intraband transitions, Au_s‑sp1_ and Au_s‑sp2_.

Using IR spectroscopy, we sought to address the nature of chemical bonding after the uptake of oxygen into the photooxidized nanocluster. IR spectra were collected for native Au_20_(SG)_16_ and self-photooxidized Au_20_(SG)_16_ in D_2_O (Figure 3). D_2_O solvent O–D stretching and bending vibration were observed at ∼2500 cm^–1^ and ∼1200 cm^–1^, respectively, similar to that reported previously.^59^ For Au_20_(SG)_16_, Figure 3A shows amide N–H bending (in-plane) and C–N stretching vibrations for the glutathione at ∼1400–1500 cm^–1^, which is consistent with literature on Au nanoclusters with peptide ligands.^60^ Figure 3A (inset *a*) shows no evidence of peroxy O–O or sulfoxy S–O bonds prior to photooxidation. Figure 3B (inset *b*) points to the presence of the O–O peroxy and S–O bonds with stretching frequencies between 865 and 1103 cm^–1^. In Figure 3B (inset *b*), we observe peaks at 865–933 cm^–1^, 952–1038 cm^–1^, and 1051–1103 cm^–1^. These observed peaks at 865–933 cm^–1^ and 952–1038 cm^–1^ are similar to those of organic peroxides, such as the reported O–O stretching frequencies of dimethyl peroxide, diethyl peroxide, and dibutyl peroxide at 890, 934, and 985 cm^–1^, respectively.^61^ Our observed peaks at 865–933 cm^–1^ are also similar to the O–O stretching frequencies of peroxo-digold complexes, (C^N^C)­AuO–O^*t*^Bu and (C^N^C)­AuO–OAu­(C^N^C) {(C^N^C) = [2,6-(C_6_H_3_^*t*^Bu-4)_2_pyridine]^2–^} which show the O–O stretching region, at 823–878 cm^–1^,^62^ and O–O stretching frequencies of Au–O_2_, Au–OOH, and Au_2_O_3_ species (840–939 cm^–1^) formed from oxidized Au atoms on the surface of Au electrodes.^63^ We do not attribute our results to M–S­(=O)_2_R (metal S-sulfinate) or M–S­(=O)­OR (metal oxysulfinyl), where peaks come mainly from S=O at 1150–1200 cm^–1^ (M = Fe, Mo, Mn, Re, Cr, Ni, Hg, and Sn), although arguably Au–O–S­(=O)­R (Au O-sulfinate) is in the range from 836 to 1048 cm^–1^.^64^ We do not attribute our data to Au–S­(=O)_2_R (Au S-sulfinate), which typically arise at 1150–1270 cm^–1^,^64^ nor to metal sulfinate, since an S=O stretch typically located at 1150–1200 cm^–1^,^65,66^ nor to a bridging O,O-bound sulfinate typically located at 950 cm^–1^ and 980 cm^–1^,^67^ all of which were not observed. Interestingly, a previous report found evidence of a bridging O,O-bound sulfinate in the photooxidation of a cadmium–sulfur cluster; here, strong intensity peaks were found at 984 cm^–1^ and 942 cm^–1^.^68^ We do observe a peak consistent with the S–O at 836–1000 cm^–1^. Literature in organosulfur chemistry indicates that S–O stretching frequencies are ∼752–875 cm^–1^ for sulfates [R–S­(=O)_2_–OH], ∼780 cm^–1^ for sulfonic esters [R–S­(=O)_2_–O–R], ∼850–950 cm^–1^ for sulfinic esters [R–S­(=O)–O–R], ∼860 cm^–1^ for sulfinic acids [R­(HO)­S=O], ∼890 cm^–1^ for sulfenic esters (R–S–O–R), ∼900 cm^–1^ for sulfonic acids [R­(HO)­S­(=O)_2_], and ∼720–690 and 760–730 cm^–1^ for sulfites [(RO)_2_S=O].^69^ Thus, we find evidence for the presence of Au–O–O–S linkage in our Au_20_(SG)_16_ postphotooxidation based on IR data for O–O and with S–O bonds similar to those found in the literature.^62–64,69^

**Figure 3:**
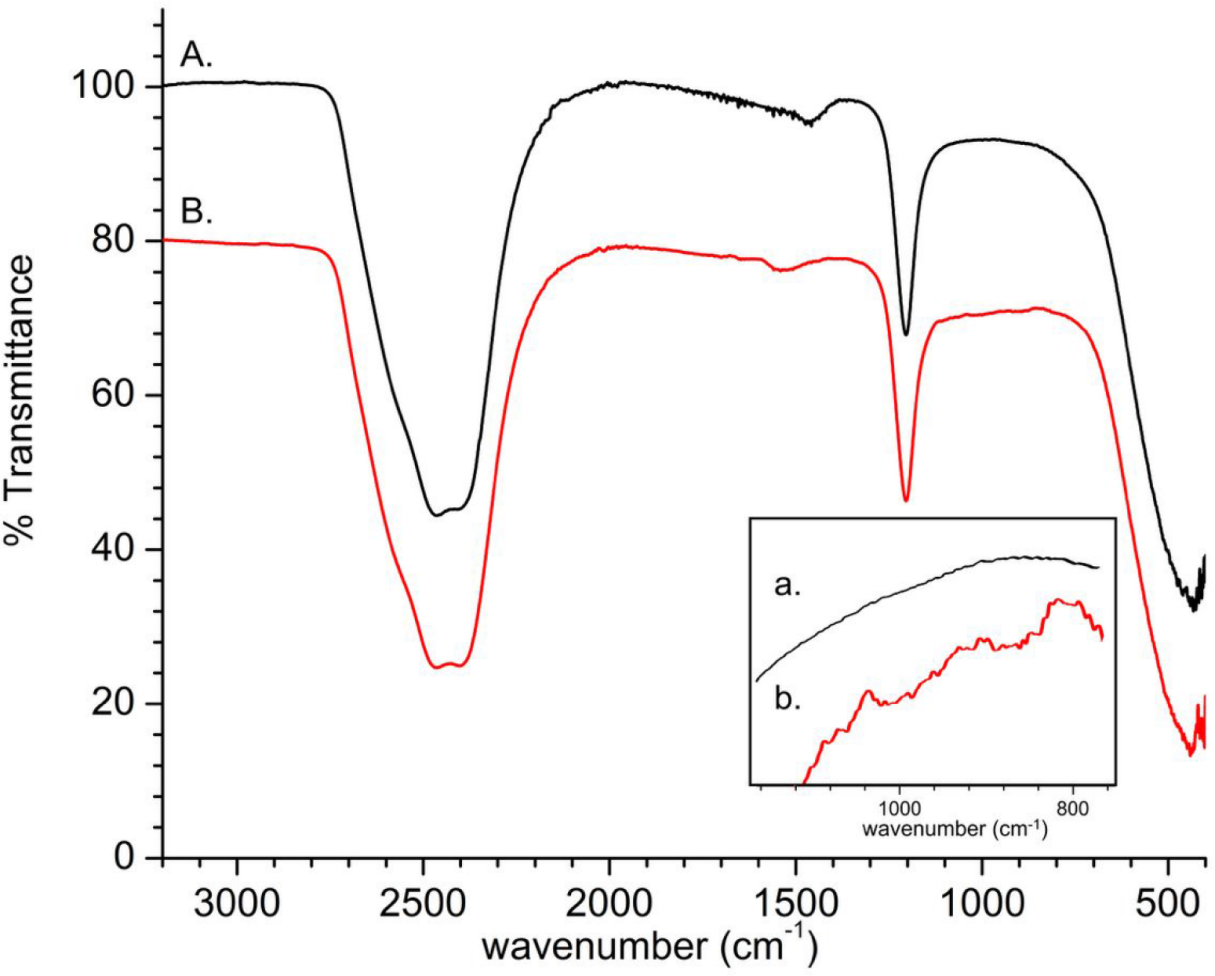
IR spectra of Au_20_(SG)_16_ (0.1 mM) prior to (A) and after self-photooxidation (B) in D_2_O. From D_2_O, O–D stretching and bending vibration observed at ∼2500 cm^–1^ and ∼1200 cm^–1^ respectively. In the glutathione ligand of Au_20_(SG)_16_, the amide N–H bending (in-plane) and C–N stretching vibrations are observed at ∼1500 cm^–1^. Inset *a* shows no evidence of the presence of the O–O peroxy and S–O bonds prior to photooxidation; inset *b* points to the presence of the O–O peroxy and S–O bonds with peaks between 865 and 1103 cm^–1^.

Next, we used DFT with M062X/GENECP [M062*X*/6-31G­(d) and LANL2DZ] to examine the type of bonding arising in the model segments of the Au nanocluster self-photooxidation reaction. We first established that AuSCH_3_ and AuS­(CH_3_^+^)Au optimize to starting structures for the addition of ^1^O_2_ (Figure 4). In the latter case, AuS­(CH_3_^+^)Au was optimized, where the thiolate moiety bridges to two Au atoms per sulfur atom. Addition of ^1^O_2_ to AuSCH_3_ and AuS­(CH_3_^+^)Au did not optimize to persulfoxide [Au–S­(O^+^–O^–^)­CH_3_ or Au–S­(O^+^–O^–^)­Au^+^CH_3_] for any start geometry that we examined to date. Instead, the starting geometries of AuSCH_3_ and AuS­(CH_3_^+^)Au with ^1^O_2_ led to dioxygen insertion to reach Au peroxides [Au–O–O–SCH_3_ and Au–O–O–SAu­(CH_3_^+^)] or to the dissociation of ^1^O_2_ away from AuSCH_3_ and AuS­(CH_3_^+^)­Au, which points to the potential for Au_20_(SG)_16_ self-photooxidation reactions to lead to Au peroxide intermediates and also physically quench ^1^O_2_. We also examined the role of postirradiation trapping of the Au_20_(SG)_16_ self-photooxidation reaction described next and consistent with a Au peroxide intermediate and oxygen-transfer process.

**Figure 4:**
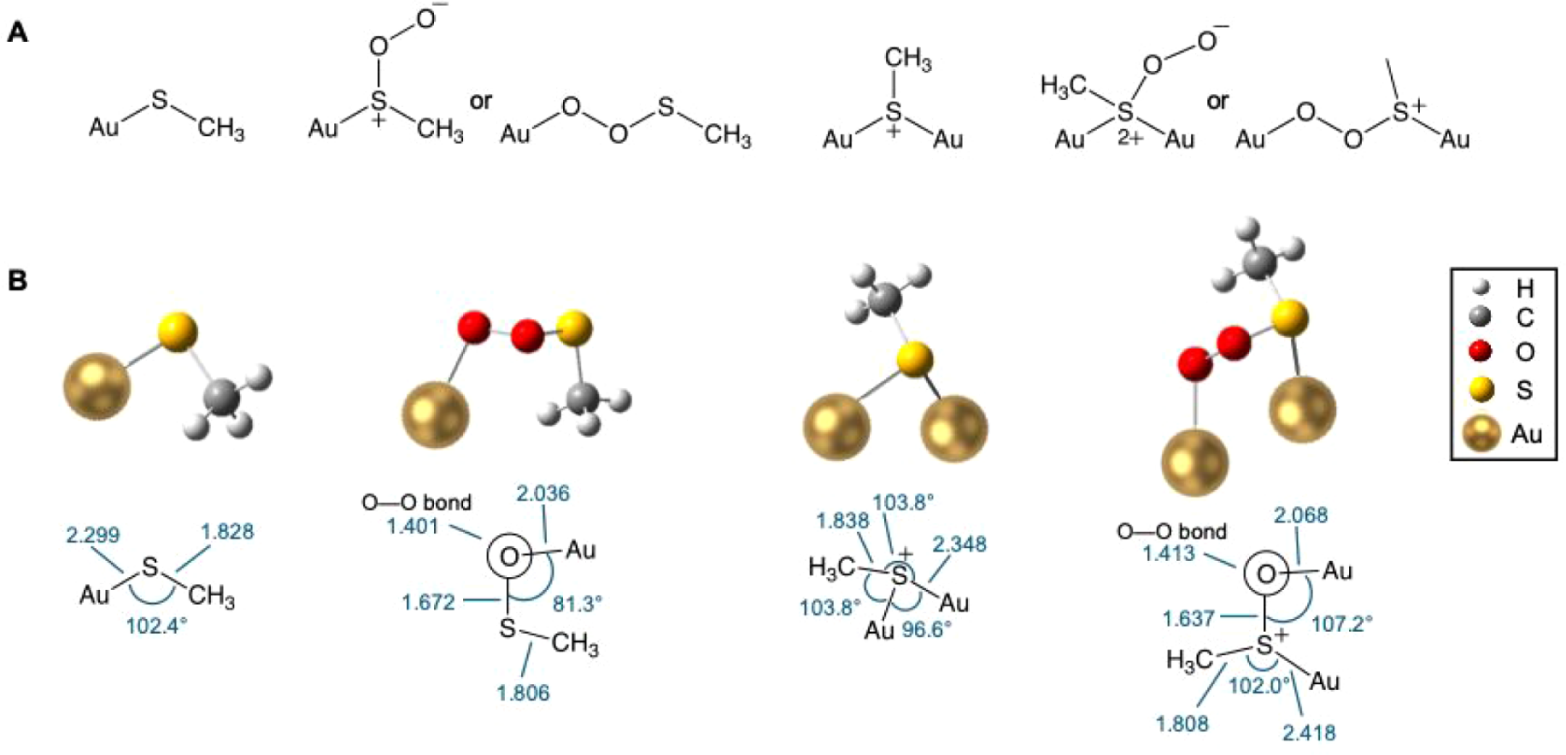
(A) Start guess geometries and (B) DFT optimized structures. Bond distances in Å; bond angles in degrees; and dihedral angles in degrees.

Finally, for further evidence on whether Au_20_(SG)_16_ undergoes self-photooxidation, we carried out a peroxide trapping study with 3-(diphenylphosphino)­benzenesulfonate anion (**1**) (Figure 5). The use of a water-soluble trapping agent such as **1** is effective for the facile detection of peroxides by ^31^P NMR. We studied the self-photooxidation of Au_20_(SG)_16_ and also the photooxidation of GSH with the AlPcS_4_ sensitizer in oxygen-saturated D_2_O. After the samples were irradiated for 1 h, the (400 < λ < 700 nm) light was turned off, and under subdued room light, samples were individually mixed with **1**. We observed oxygen-atom transfer with the photooxidized sample of Au_20_(SG)_16_ (a 99-fold greater conversion of phosphine **1** to phosphine oxide **2**) compared to the photooxidized sample of GSH. These results pointed to the presence of peroxides of ≥40 μM in the former but less than 0.4 μM in the latter. Further, the trapping data point to the formation of a Au–O–O–SG instead of a hydroperoxide species (e.g., Au–O–O–H) species based on the ^1^H NMR data obtained. ^1^H NMR spectrum of the self-photooxidation of Au_20_(SG)_16_ showed no new peaks from 9.5 to 12 ppm, suggesting the absence of hydroperoxide OOH peaks.

**Figure 5:**
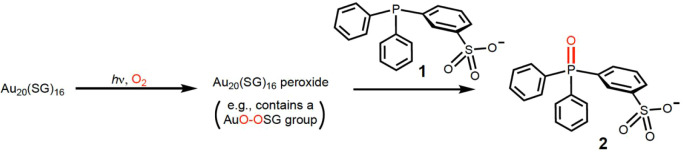
Detection of Au_20_(SG)_16_ peroxides by the conversion of phosphine **1** to phosphine oxide **2** in the dark using ^31^P NMR spectroscopy.

### Mechanistic Considerations

We provide evidence for the proposed mechanism in Figure 6. Au_20_(SG)_16_ is found to serve dual roles as a photosensitizer for ^1^O_2_ formation and a quencher of ^1^O_2_. We found that (i) Photoexcitation of Au_20_(SG)_16_ produces the 1270 nm ^1^O_2_ signal. The *k*_T_ for ^1^O_2_ removal by Au_20_(SG)_16_ (∼1–2 × 10^8^ M^–1^ s^–1^) was 11- to 22-fold greater than that of GSH itself. This result points to thiolate content as key to ^1^O_2_ quenching, with the Au atoms playing a less significant role in the ^1^O_2_ quenching. Relatedly, the *k*_T_ value for Au_20_(SG)_16_ bears a similarity to other photosensitizers; it is lower than methylene blue (MB) (4 × 10^8^ M^–1^ s^–1^), but higher compared to rose bengal (1.5 × 10^7^ M^–1^ s^–1^) and tetraphenylporphyrin (4.4 × 10^7^ M^–1^ s^–1^).^70^ Furthermore, there are similarities of Au_20_(SG)_16_’s *k*_T_ value to Au_39_(2-phenylethanethiolate)_29_ (1.1 × 10^7^ M^–1^ s^–1^)^71^ and Au_25_[S­(*n*-C_4_H_9_)]_18_^–^ (3.4 × 10^8^ M^–1^ s^–1^),^72^ as well as Au­(I) thiolate metal complexes, including auranofin (1-thio-β-glucopyranosato)­(triethylphosphine)­gold­(I) 2,3,4,6-tetraacetate) (0.2 × 10^7^ M^–1^ s^–1^) and 1-methylthio­(triethylphosphine)­gold­(I) (3.7 × 10^7^ M^–1^ s^–1^) as reported in review articles by Selke and coworkers.^73,74^ Au_20_(SG)_16_ is similar to Au thiolate compounds reported by Selke and coworkers, which also produce a 1270 nm ^1^O_2_ signal upon photoexcitation.^73^ In terms of self-photooxidation, Au_20_(SG)_16_ bears a similarity to other ^1^O_2_ photosensitizers, such as porphyrins and xanthenes, in the initial formation of peroxides.

**Figure 6:**
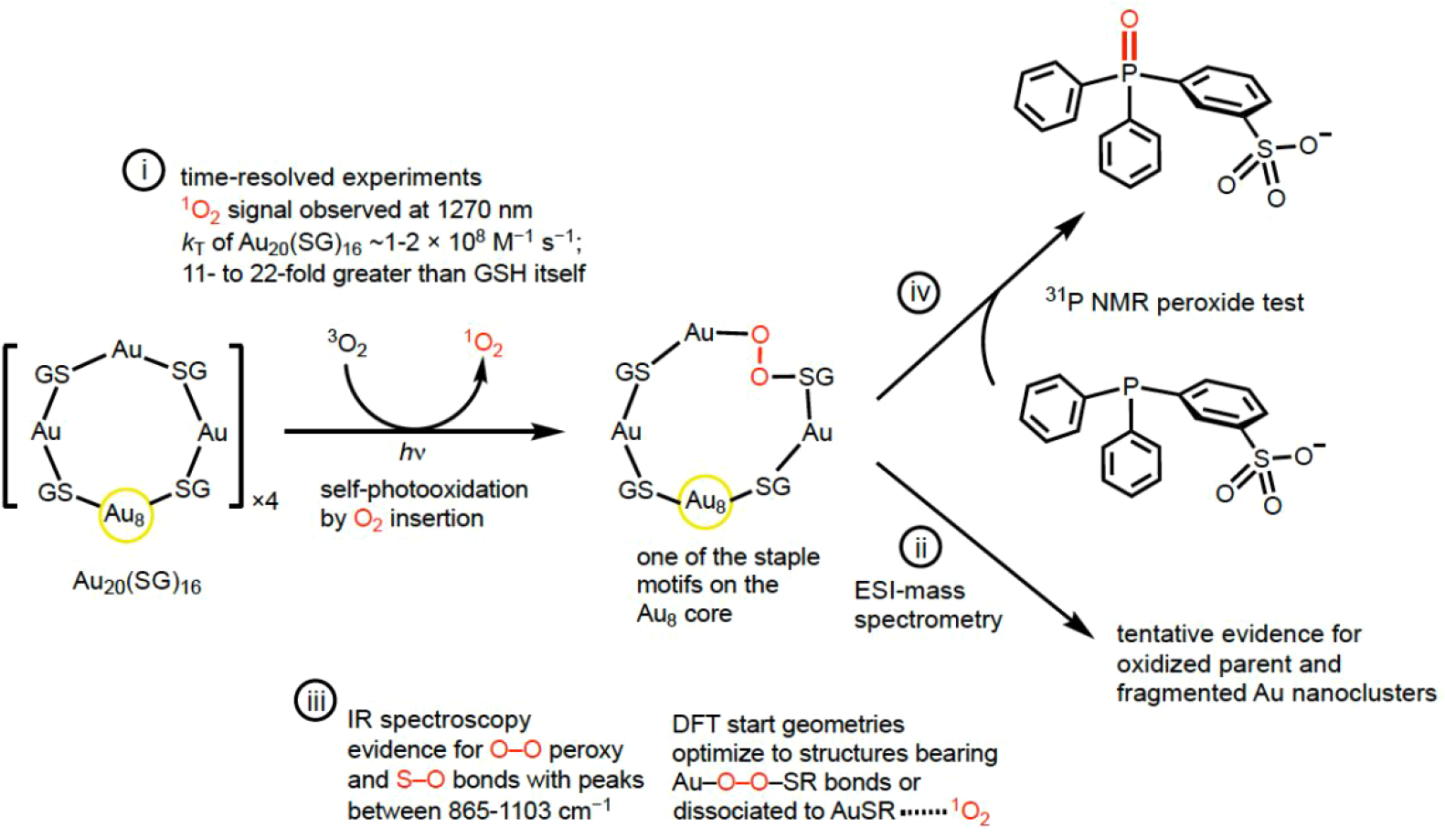
Mechanistic summary of the self-sensitized formation of ^1^O_2_ and self-photooxiation of Au_20_(SG)_16_ leading to Au peroxides.

(ii and iii) We gained insight into the structure of the self-photooxidized Au_20_(SG)_16_ nanocluster. ESI-MS, IR, DFT, and phosphine trapping results were useful. ESI-MS results indicated that Au_20_(SG)_16_ self-photooxidizes, taking up dioxygen to the parent compound and cleaving into smaller oxygenated clusters of undetermined structures. IR results of the self-photooxidized Au nanocluster point to the presence of O–O peroxy and S–O bonds, which are attributed to a Au nanocluster peroxide with Au–O–O–SG units. Such results were similar to those of peroxo-digold complexes and Au–O–O–H compounds,^62,63,75,76^ and organic peroxides, but inconsistent with metal *S*-sulfinate and metal oxysulfinyl compounds. The dioxygen uptake in Au_20_(SG)_16_ is also reminiscent of other photosentizers, such as self-photooxidized PpIX producing endoperoxides with latent decomposition (Scheme 2C).^48^ DFT was also used to compute interactions of ^1^O_2_ with model compounds AuSCH_3_ and AuS­(CH_3_^+^)Au to rationalize when Au thiolate peroxide formation can take place. Optimization to Au persulfoxides, e.g., Au–S­(O^+^–O^–^)­R, proved to be elusive. When optimizing the structures, an interesting facet is that ^1^O_2_ is drawn to and inserts into the Au–SR bond. Various starting geometries led to the facile optimization to Au peroxides [Au–O–O–SCH_3_ and Au–O–O–SAu­(CH_3_^+^)]. Such Au peroxides can be expected to be sufficiently long-lived for trapping postirradiation unlike short-lived candidate intermediate Au persulfoxide or other intermediates similar to those in organic sulfide/^1^O_2_ reactions.^77^ Control experiments with sodium 3-(4-methylnaphthalene-1-yl-propanoate) (100 mM), a chemical source of ^1^O_2_ in D_2_O solutions of Au_20_(SG)_16_ (0.1 mM) were carried out, but did not reveal ROOH protons, also consistent with our proposal of a Au–O–O–SG product.

(iv) We propose the insertion of dioxygen into the nanocluster, which destabilizes the structure to break into smaller nanoclusters but is suitable to be trapped with a phosphine trapping agent. Phosphine **1** served as a peroxide test for the O atom transfer to corresponding phosphine oxide **2** and detection by ^31^P NMR. Phosphine **1** is water-soluble and sufficiently nucleophilic at P despite having an electron-withdrawing sulfonate anion on one of the three aromatic rings. Phosphine **1** added postirradiation to photooxidized samples of Au_20_(SG)_16_ and GSH showed transfer of O atoms to a much greater extent in the former. No peroxides in any significant concentration were detected in the photooxidized sample of GSH. Previous reports^62^ have also used triaryl phosphines as peroxide tests for Au–O–O–H, Au–O–O–Au, and other compounds.

In summary, there is photosensitized formation of ^1^O_2_ by Au_20_(SG)_16_. But there is rapid total quenching of ^1^O_2_ by ground-state Au_20_(SG)_16_ at higher concentrations of the nanocluster based on time-resolved studies. There are competitive paths to nanocluster quenching of ^1^O_2_, one is physical quenching of ^1^O_2_ to ^3^O_2_, and the other is chemical quenching. The latter appears to be a minor path but over time leads to dioxygen insertion. Au–O–O–SG bonding is attributed to the chemical quenching path based on the results from IR, ESI-MS, DFT, and phosphine trapping studies. The expansion of the staple from dioxygen insertion with its O–O group decreases the nanocluster stability compared to unoxidized Au_20_(SG)_16_.

## Conclusion

Mechanistic questions were probed to improve our understanding of Au_20_(SG)_16_ self-photooxidation. Questions were probed with photophysical, photochemical, theoretical, and indirect trapping methods. Using these methods together was advantageous, since studies of photosensitizers undergoing self-photooxidation, often referred to as photobleaching, are usually ill-defined or noted only as artifacts in microscopy fields rather than interesting and potentially useful peroxide-forming features.

It could be argued that the PDT activity will be enhanced by the postirradiation reaction of the Au nanocluster peroxide due to a longer lifetime than the initially formed reactive oxygen intermediates, such as ^1^O_2_. There are no available data on the downstream decomposition products from self-photooxidation Au nanoclusters. Detection of AuO**•** radicals or other peroxide homolysis products presents challenges. Also, our conclusion is that the thiolates are key sites for ^1^O_2_ attack; an appropriate conclusion since ^1^O_2_ quenching by Au_20_(SG)_16_ was nearly proportional to the GSH content, some 11–22 times greater than glutathione itself.

Future Au nanocluster designs could include (i) fluorinated thiolate ligands to protect the system from self-photooxidation. A fluorinated sensitizer on a superhydrophobic surface by Lyons et al. was shown to be stable to self-photooxidation.^78^ Since ligands are tailorable, a balance of ^3^O_2_ access for Dexter energy transfer (not too congested), but also offering a protection shell, will require a give-and-take between the two. (ii) In view of the evidence of Au–O–O–SG bonding from ^1^O_2_ insertion into Au_20_(SG)_16_, experiments where the temperature can be lowered would be expected to increase the stability of the gold nanocluster peroxide. Even temperatures near 0 °C in water would be available to exploit techniques such as low temperature FAB mass spectrometry.^79,80^

## Supplementary Material



## Notes

Information about the synthesis and characterization of Au_20_(SG)_16_, ^1^O_2_ luminescence decay curves, HPLC, ESI-MS, IR, and ^31^P NMR data of self-photooxidized Au_20_(SG)_16_, and DFT computed geometries and energetics (PDF)
