# Supplementary material for: “Blebbing” of a Gold Nanocluster by Dioxygen Insertion into Thiolate Staples during Self-Photooxidation
Source: J Phys Chem A. 2025 Dec 12;129(51):11810–9. doi: 10.1021/acs.jpca.5c06629 (PMC12746443; doi:10.1021/acs.jpca.5c06629)
Supplement: Supplementary file 1 [file jp5c06629_si_001.pdf]

Supporting Information

"Blebbing" of a Gold Nanocluster by Dioxygen Insertion into Thiolate Staples during Self-Photooxidation

Serah Essang[1,2] and Alexander Greer[1,2]*

[1] Department of Chemistry, Brooklyn College, Brooklyn, New York, USA

[2] Ph.D. Program in Chemistry, The Graduate Center of the City University of New York, New York, New York, USA

Table of Contents

Page Number

S2	Description of Synthesis and Characterization of $Au_{20}SG_{16}$.	
S3	Figure S1:	HPLC trace of $Au_{20}SG_{16}$.
S3	Figure S2:	ESI-MS spectrum of $Au_{20}SG_{16}$.
S4	Table S1:	ESI-MS data for $Au_{20}SG_{16}$.
S4	Figure S3:	$^1O_2$ luminescence decay curves in the presence of $Au_{20}SG_{16}$ or GSH.
S5	Figure S4:	ESI-MS spectra of self-photooxidized $Au_{20}SG_1$.
S6	Table S2:	ESI-MS and retention time information of $Au_{20}SG_{16}$ and self-photooxidized $Au_{20}SG_{16}$.
S6	Figure S5:	IR spectra of $Au_{20}SG_{16}$ before and after self-photooxidation.
S7	Figure S6:	^{31}P NMR spectra of phosphine **1** and its oxide **2** after trapping an O-atom from self-photooxidized $Au_{20}SG_{16}$.
S8	Figure S7:	DFT computed geometries and energetics.

Synthesis and Characterization. Figure S1 shows HPLC of the purified $Au_{20}SG_{16}$. The HPLC trace shows that we have a major peak that corresponds to $Au_{20}SG_{16}$. The inset shows the PAGE separation and the $Au_{20}SG_{16}$ nanocluster, this was found and isolated from the top black band. Lower bands were not isolated.

Figure S2 shows the ESI-MS spectrum of purified $Au_{20}SG_{16}$ shown in HPLC trace and the band in the PAGE shown in Figure S1. Table S1 shows that the ESI-MS spectrum shows *m/z* 1768.3102 with charge z = 5 corresponding to a deconvoluted mass of 8841.5510 which is in agreement with the predicted mass of cluster ion $[Au_{20}SG_{16} + 5H]^{5+}$, *m/z* 2210.1401 with charge z = 4 corresponding to a deconvoluted mass of 8840.5604 which is in agreement with the predicted mass of cluster ion $[Au_{20}SG_{16} + 4H]^{4+}$, *m/z* 2946.5100 with charge z = 3 corresponding to a deconvoluted mass of 8839.5300 which is in agreement with the predicted mass of cluster ion $[Au_{20}SG_{16} + 3H]^{3+}$.

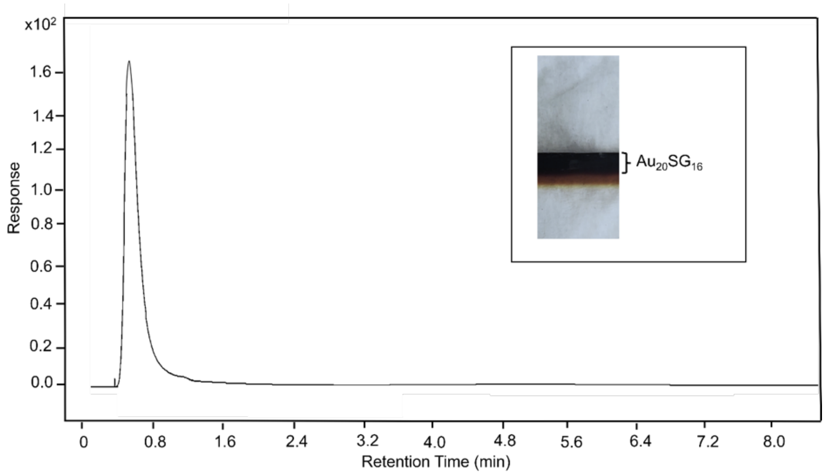

Figure S1. HPLC trace of $Au_{20}SG_{16}$. The inset shows PAGE where the $Au_{20}SG_{16}$ is located; it is isolated from the top black band, lower bands were not isolated.

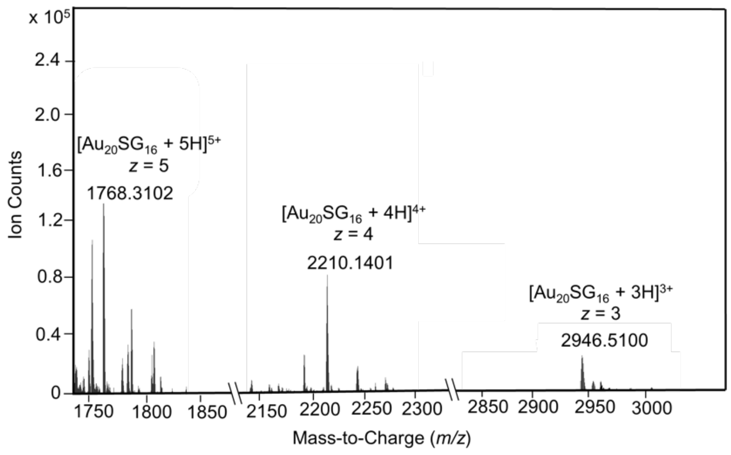

Figure S2. ESI-MS spectrum of $Au_{20}SG_{16}$.

Table S1. ESI-MS data for $Au_{20}SG_{16}$.

Cluster ion	Charge State (*z*)	Experimental *m/z*	Experimental Monoisotopic Mass	Calculated Monoisotopic Mass
$[Au_{20}SG_{16} + 5H]^{5+}$	+5	1768.3102	8841.5510	8841.5859
$[Au_{20}SG_{16} + 4H]^{4+}$	+4	2210.1401	8840.5604	8840.5780
$[Au_{20}SG_{16} + 3H]^{3+}$	+3	2946.5100	8839.5300	8839.5702

[a] The charge has been deduced from the spectra, e.g., m/z was multiplied by the charge (+3 or +4 or +5) to give the experimental mass.

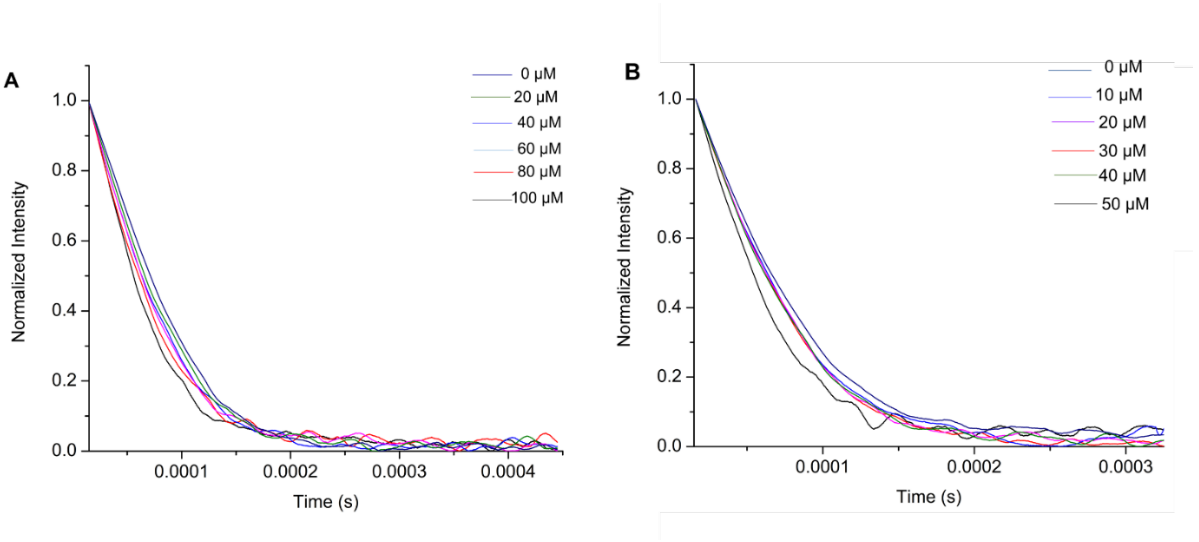

Figure S3. Representative normalized decay curves for the $^1O_2$ luminescence at 1270 nm in D_2O with increasing concentrations of (A) $Au_{20}SG_{16}$, and (B) GSH in the presence of $AlPcS_4$ (50 μM).

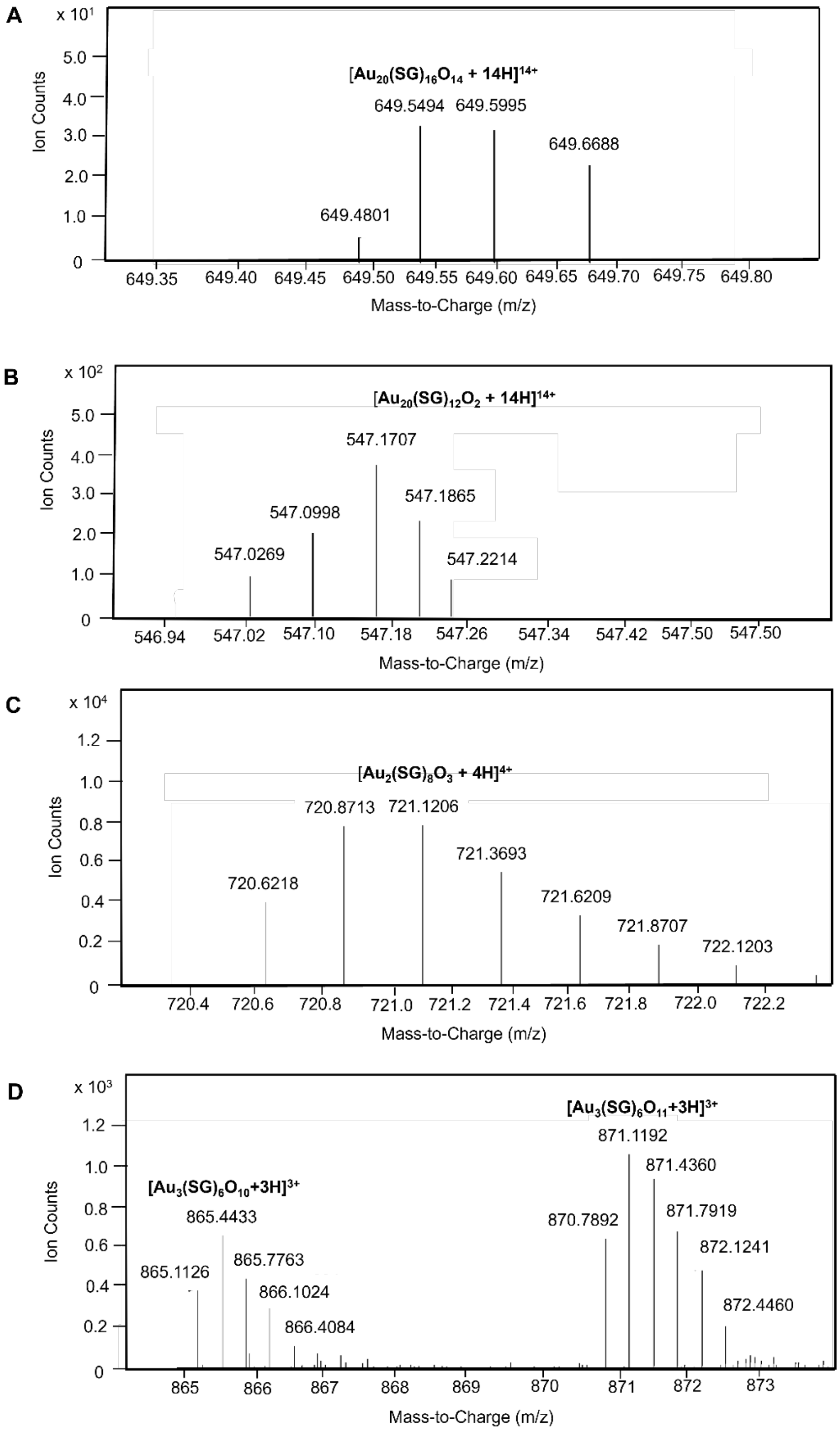

Figure S4. ESI-MS spectra of self-photooxidized $Au_{20}SG_{16}$. (A) MS data consistent with $[Au_{20}(SG)_{16}O_{14} + 14H]^{14+}$, (B) MS data consistent with $[Au_{20}(SG)_{12}O_2 + 14H]^{14+}$ (C) MS data consistent with the $Au_2(SG)_8O_3$ fragment, and (D) MS data consistent with the $Au_3(SG)_6O_{10}$ and $Au_3(SG)_6O_{11}$.

Table S2. ESI-MS and retention time information on unoxidized $Au_{20}SG_{16}$ and self-photooxidized $Au_{20}SG_{16}$ in D_2O

	Cluster ion	Retention time (min)
unoxidized	$[Au_{20}SG_{16} + 4H]^{4+}$	0.9-1.0
after photooxidation	$[Au_{20}(SG)_{16}O_{14} +14H]^{14+}$	3.7-3.9
	$[Au_{20}(SG)_{12}O_2 +14H]^{14+}$	3.7-3.9
	$[Au_2(SG)_8O_3 + 4H]^{4+}$	2.9-3.4
	$[Au_3(SG)_6O_{11} + 3H]^{3+}$	5.7-7.3
	$[Au_3(SG)_6O_{10} + 3H]^{3+}$	5.7-7.3

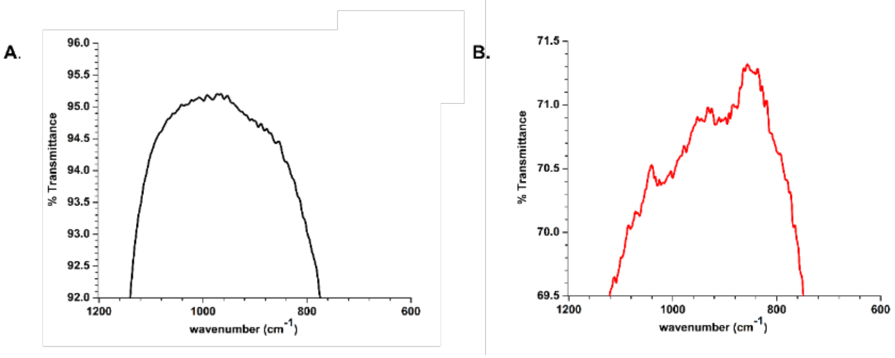

Figure S5. IR spectra of $Au_{20}SG_{16}$ in the range 1200-600 cm^{-1}, before (A) and after (B) self-photooxidation, respectively.

The quantification procedure used to obtain the reported conversion and concentration values is as follows:

$$Conversion\ Concentration = \frac{Integral\ of\ Oxidized\ Phosphine\ Peak}{Integral\ of\ Unreacted\ Phosphine\ Peak + Integral\ of\ Oxidized\ Phosphine\ Peak} \times Molarity\ of\ Solution$$

To obtain the percent conversion, instead of multiplying by the molarity of solution in the above equation, we multiplied the value by 100.

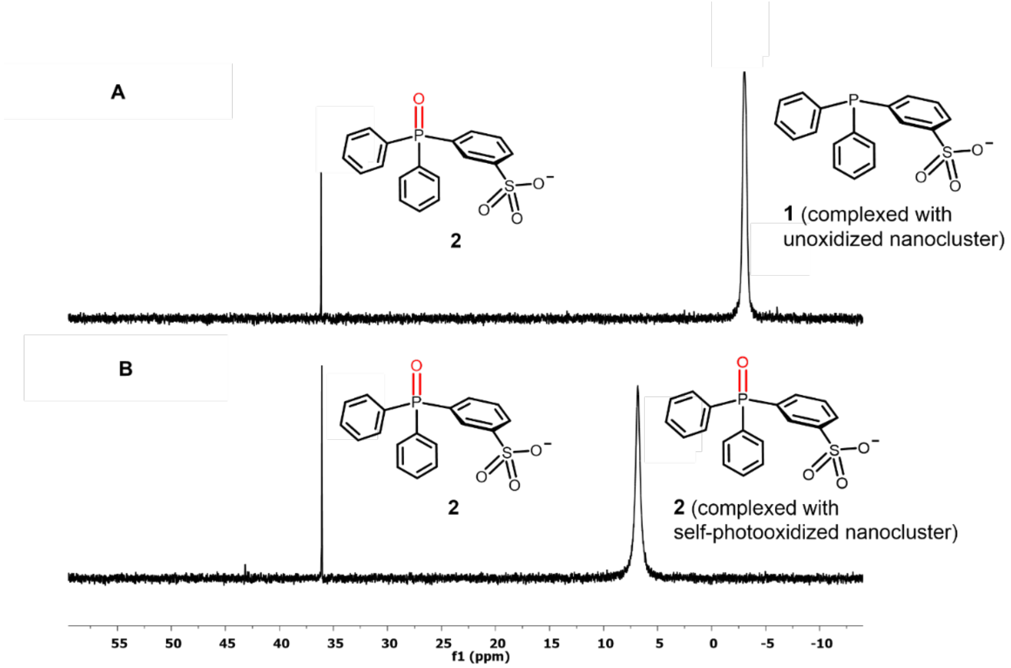

Figure S6. (A) ^{31}P NMR spectrum of phosphine **1** and $Au_{20}SG_{16}$ prior to irradiation in D_2O. The phosphine **1** peak at -6 ppm broadens and shifts to -3 ppm for due to its association with $Au_{20}SG_{16}$ with phosphine oxide **2** present as an impurity (1.3%). (B) ^{31}P NMR spectrum of phosphine oxide **2** after the self-photooxidation of $Au_{20}SG_{16}$ in D_2O. The phosphine oxide **1** peak located at 37 ppm appears at 6.8 ppm with broadening due to its association with oxidized $Au_{20}SG_{16}$.

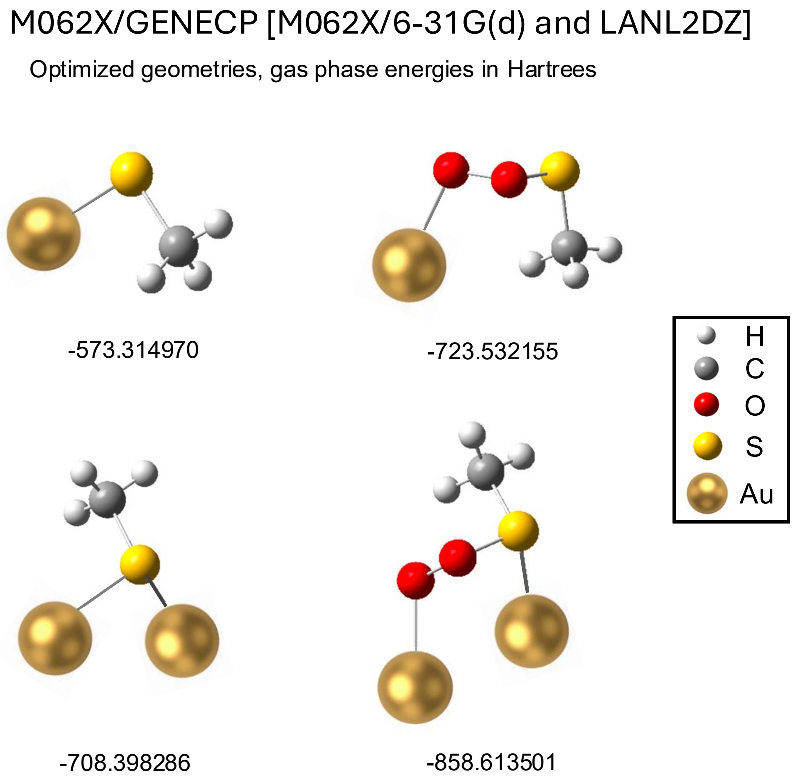

Figure S7. DFT computed geometries and energetics.
